# The case for homebrew AI in diagnostic pathology

**DOI:** 10.1002/path.6438

**Published:** 2025-07-04

**Authors:** Julien Calderaro, Helen Morement, Frédérique Penault‐Llorca, Stephen Gilbert, Jakob Nikolas Kather

**Affiliations:** ^1^ Département de Pathologie Assistance Publique Hôpitaux de Paris, Groupe Hospitalier Henri Mondor Créteil France; ^2^ MINT‐HEP Mondor Integrative Hepatology, Institut Mondor de Recherche Biomédicale Université Paris Est Créteil Créteil France; ^3^ AMMF‐The Cholangiocarcinoma Charity Chelmsford UK; ^4^ INSERM U1240, [Molecular Imaging & Theragnostic Strategies (IMOST)] University Clermont Auvergne Clermont‐Ferrand France; ^5^ Department of Pathology Centre Jean PERRIN Clermont‐Ferrand France; ^6^ Else Kröner Fresenius Center for Digital Health TUD Dresden University of Technology Dresden Germany; ^7^ Department of Medicine University Hospital Dresden Dresden Germany; ^8^ Medical Oncology National Center for Tumor Diseases, University Hospital Heidelberg Heidelberg Germany

**Keywords:** artificial intelligence, pathology, deep learning, whole‐slide image, laboratory‐derived test, homebrew

## Abstract

Artificial intelligence (AI) methods for digital pathology have tremendous potential to improve cancer diagnostics, biomarkers, and ultimately patient care. These AI methods, if marketed and sold, require authorisation or clearance as *in vitro* diagnostic (IVD) devices by regulatory bodies like the Food and Drug Administration (FDA) in the USA or Notified Bodies in the European Union (EU). Many AI tools for digital pathology are unlikely to be commercially viable and taken up by commercial entities ready to navigate these complex and costly processes. However, a longstanding quality framework already exists that allows for lab‐developed tests, colloquially known as ‘homebrew’ tests, that are locally validated and performed under the responsibility and oversight of the pathologist. Here we argue for advancing homebrew AI systems within this existing framework to enhance patients' access to supportive digital diagnostic tools. We outline how homebrew AI models are currently permitted under regulatory provisions in the USA and the European Union, how a new US FDA rule may effectively regulate them out of existence, and propose steps to facilitate the safe and effective integration of homebrew AI models in pathology practice. © 2025 The Author(s). *The Journal of Pathology* published by John Wiley & Sons Ltd on behalf of The Pathological Society of Great Britain and Ireland.

## Background

Artificial intelligence (AI) will fundamentally advance medical practice by improving diagnostic precision, guiding treatment selection, and performing routine clinical tasks automatically [1]. Pathology, in particular, stands at the forefront of AI innovations due to the suitability of deep‐learning techniques for image analysis [1]. The number of AI‐related pathology publications is increasing exponentially, and substantial investments have been made by companies and hospitals to digitize workflows, modernize information systems, and develop AI‐based diagnostic assistants and biomarkers. Current AI models primarily assist with tasks considered repetitive or tedious, such as screening lymph nodes for metastasis or identifying tumour foci on prostate biopsies [2]. These AI‐based solutions are classified as (*in vitro* diagnostic) medical devices, necessitating authorisation or clearance from regulatory institutions such as the Food and Drug Administration (FDA) in the USA or Notified Bodies in the European Union before they can be marketed [3]. These regulatory requirements create significant barriers to bringing AI solutions into clinical practice.

Modern pathology combines multiple diagnostic methods to reach accurate diagnoses. The pathological diagnostic process is inherently complex, involving multiple sequential stages. Haematoxylin‐eosin (H&E) staining provides an initial analysis of cell structures and tissue architecture, but additional techniques like special staining or immunohistochemistry (IHC) are often required to refine diagnoses, predict prognosis, or determine the origin of metastases. These supplementary techniques are performed in pathology departments and are not typically CE marked (the EU regulatory approval mark) or FDA‐approved as commercial medical devices [3]. Instead, they are considered ‘homebrew’ tests, developed and validated within individual laboratories.

Quality control is central to the successful implementation of homebrew tests in pathology. Despite lacking formal regulatory approval, these tests undergo individual lab‐based assessments essential for laboratory accreditation. Pathologists and laboratory technicians oversee these processes, defining quality criteria for each technique [3]. Control tissues with known outcomes are analysed alongside patient samples to ensure accuracy and reliability under the laboratory's specific conditions [3]. For instance, in HER2 IHC – a common and highly relevant test to determine eligibility for anti‐HER2 therapy in breast cancer patients – control tumour tissues with established HER2 statuses are included on the same slide as the patient sample [4]. Pathologists review these controls to confirm expected staining patterns and intensities, ensuring the test's validity. The widespread use of homebrew tests in cancer pathology demonstrates their established role in clinical practice.

Understanding the limitations of diagnostic tests is fundamental to their appropriate use in clinical practice. Most of these techniques are neither 100% sensitive nor 100% specific. For example, CDX2 expression, used to identify colorectal origin in metastatic tumours, may be negative in up to 5% of colorectal cancers and positive in a subset of adenocarcinomas from other organs like the ovary, bile ducts, stomach, or urinary bladder [5, 6, 7]. Other markers such as TTF1, GATA3, or PAX8 have similar limitations but remain invaluable in diagnostic workflows. Pathologists are acutely aware of these potential pitfalls. They integrate results from multiple complementary techniques, considering various converging pieces of evidence to reach a final histological diagnosis. Through rigorous internal validation and external quality assessment, pathology departments ensure reliable diagnostic results despite these inherent limitations.

## The role of homebrew AI in pathology practice

Pathologists routinely perform semiquantitative evaluations of tissue samples, including assessments of fibrosis and steatosis in liver biopsies and measurements like the percentage of Ki‐67‐positive tumour cells in neuroendocrine tumours. These evaluations are inherently subjective and show considerable variation between different observers. Homebrew AI models can address these challenges by providing standardized quantitative measurements of clinically relevant features. Most of these models generate prediction heatmaps that are overlaid directly onto digital slides, allowing pathologists to verify the AI's analysis using their professional judgement. While current AI models have some limitations in explaining their decisions through heatmaps [8], pathologists can draw on their medical training and experience to properly interpret and validate these results, similar to how they interpret traditional homebrew IHC tests that have been used for over 80 years.

We argue that noncommercial, homebrew AI models can be advanced and integrated into pathology practice, much like other homebrew tests have been. Pathologists should have the autonomy to select and develop models suited to their specific needs, overseeing their validation and implementation. By applying the same local validation and quality control processes used for traditional homebrew tests, these AI models can become valuable tools in enhancing diagnostic accuracy and efficiency. Numerous promising AI models have been developed in academic settings. Unfortunately, many may never be implemented in clinical practice due to regulatory and commercialization barriers. By embracing the homebrew approach, pathologists can evaluate these models within their laboratories, determining their performance in their unique technical environments and identifying potential biases or limitations. This enables pragmatic, reasoned, and transparent integration — or exclusion — of AI model predictions into final diagnoses.

Advancing homebrew AI systems is particularly crucial for rare diseases and cancers, where small market sizes discourage commercial development. Patients with these conditions stand to benefit significantly from AI advancements. Without the flexibility to develop and implement homebrew AI models, these patients may lack access to innovative diagnostic tools. Empowering pathologists to develop AI models within their departments helps ensure that AI advances reach all clinical settings equitably. Modern tools and platforms now allow pathologists without extensive computational expertise to train and deploy AI models. This wider access to AI technology supports the development of homebrew models within pathology departments, fostering innovation and improving patient care. This will probably be associated with several additional costs for departments/hospitals, which may be related to hardware, software, legal, compliance, operational expenses, and staff (AI/IT engineers). Although it is difficult to make a precise estimate of the overall amount required, it is likely that these amounts are reasonable on the scale of large hospitals. In fact, the largest part is linked to servers and their maintenance, and are already required by the departments' digital transformation.

## Risks associated with the use of homebrew AI models

Model drift is a term used in AI to describe how the performance of a model slowly gets worse over time or once implemented in practice. AI model drift in histology can arise from different factors, such as changes in the digital environment, data distribution/disease characteristics, technical and clinical workflows (scanner settings, tissue preparation, or staining protocols) or training data biases. To mitigate these risks, monitoring of model performance is critical, and retraining, fine‐tuning, and/or recalibration using more diverse and up‐to‐date datasets could also be considered.

Automated drift detection systems will be key to ensure easy, large‐scale control of the different AI models implemented in pathology departments. Another crucial task is the collection and sharing of diverse test datasets, which could be performed by pathology quality societies.

In this context, we believe that pathologists implementing and operating AI models must themselves develop adequate AI literacy to make a safe and responsible use of these models, or they must ensure that the relevant skill sets are represented in their teams. Vos *et al* recently proposed a list of skills that should be added to pathology residency programs, which include basic understanding of informatics, knowledge about the steps required to develop and validate a model, and how to critically assess a model and its implementation and recognizing potential ethical/legal issues [9]. Having clearly defined operating procedures that include periodic expert pathologist review and assessment AI systems is essential, even where automated monitoring systems are in place.

Knowledge of the best metrics to use is critical. The AUC‐ROC (area under the receiver operating characteristic curve) is one of the most popular approaches, as it provides an overview of the ability of a given model to correctly predict both positive cases and negative cases across different classification thresholds. Other metrics such as sensitivity (recall true‐positive rate), specificity (true‐negative rate), and precision (positive predictive value) could also be very useful. Accuracy, however, is generally not recommended, as it is can be misleading when dealing with imbalanced classes.

## Regulation of homebrew AI in the European Union

In the European Union, the use of homebrew tests is explicitly permitted under the In Vitro Diagnostic Medical Devices Regulation (IVDR) Article 5 (5) [10]. This provision allows health institutions to manufacture and use their own devices—including AI‐based models—provided they meet certain conditions. These conditions include that the laboratory takes on responsibility for ensuring that the devices meet relevant quality, safety, and performance requirements and are not transferred to another legal entity. An illustrative example is MLL (Münchener Leukämielabor) in Germany, one of the largest diagnostic haematology laboratories in Europe. MLL has successfully utilized this regulatory framework to develop and implement a series of AI‐enabled homebrew tests [11]. Their quality management system ensures compliance with safety and performance standards, and they have integrated AI models into their diagnostic workflows effectively. MLL's experience demonstrates the feasibility and benefits of advancing homebrew AI systems within existing regulatory provisions. In this line, several studies have also investigated the use of digital image analysis platforms to quantify Ki‐67 immunostaining in breast cancer. The reproducibility and clinical relevance of various solutions, including the open‐source software Qupath, was assessed [12, 13]. Authors observed a very high reproducibility both within and between the different platforms, and outputs were related to patients' outcomes. Although these studies are retrospective, their results are reassuring and support the use of these approaches.

The EU's Medical Device Coordination Group (MDCG) has provided guidance clarifying the application of Article 5 (5). According to MDCG 2023‐1, in‐house manufactured devices are exempt from the regulation's requirements for third‐party regulatory assessment if used within the legal entity [14]. This means that health laboratories and pathology departments within hospitals can develop and use homebrew AI models without undergoing the full conformity assessment procedures required for generally commercialized devices. The forthcoming European Union AI Act introduces additional considerations for AI systems in healthcare [15]. However, devices developed and used within health institutions, under the IVDR's homebrew provisions, are not subject to the AI Act's high‐risk system requirements in the same way as commercial devices. Consequently, homebrew AI models can continue to be developed and used in pathology departments, provided the laboratory locally oversees compliance with necessary safety and performance conditions.

## Regulation of homebrew AI in the United States

In the United States, homebrew tests – often referred to as laboratory‐developed tests (LDTs) – have traditionally operated under the Clinical Laboratory Improvement Amendments (CLIA) of 1988. Under CLIA, laboratories could develop and use their own tests without FDA approval, operating under a policy of ‘enforcement discretion’. This framework allowed for the development and implementation of innovative diagnostic tools, including AI‐enabled tests, within clinical laboratories.

Recently, the FDA has moved to reconsider the policy of enforcement discretion for certain laboratory‐developed tests, aiming to bring them under more stringent regulatory oversight. This policy shift does not include traditional histopathology and IHC approaches, which will continue to be under enforcement discretion as ‘1976‐Type LDTs’ that have characteristics common among LDTs that were available in 1976. However, this substantially tighter regulatory framework is expected to restrict the use of homebrew AI‐enabled histopathology decision support tools.

While this increased regulatory scrutiny aims to ensure patient safety, we argue that it does not leave sufficiently wide flexibility for noncommercial pathology laboratories to exercise pragmatic medical freedom. Proponents of this tightening, including the FDA and commentators, argue that the medical‐diagnostics field has fundamentally changed and that tests are more common, used in higher‐risk settings, and are used novelly to select therapies for critically ill patients, and make use of complex proprietary algorithms [16, 17]. Histopathology IHC has been used for decades to recognise the most severe conditions and to plan treatments, and algorithms for histopathology are often open‐source rather than proprietary [18]. In our view, although the EU has often been accused of overregulating AI in medicine, in its approach to the regulation of noncommercial homebrew AI systems it is showing balanced pragmatism and restraint [19]. It is crucial for stakeholders to engage in dialogue with US regulatory agencies to develop clear pathways that balance innovation with safety, enabling the continued advancement of homebrew AI models in pathology.

## Legal challenges to the FDA's LDT rule

The FDA's proposed regulatory changes have sparked a number of legal challenges and opposition from professional organizations. On 29 May 2024, the American Clinical Laboratory Association (ACLA) filed a lawsuit in the U.S. District Court for the Eastern District of Texas, and this has been supported in a filing by the American Society for Clinical Pathology (ASCP) [20]. On 19 August 2024, the Association for Molecular Pathology (AMP), along with an individual pathologist, filed a similar lawsuit in the U.S. District Court for the Southern District of Texas. The disputes centre on the FDA's statutory authority to regulate LDTs and the outcome of these challenges will significantly impact the future development and implementation of homebrew AI models in pathology laboratories in the USA. On 31 March 2025 a judge examined both filings and decided that the FDA's rulemaking was burdensome, sided with the ACLA and AMP, and struck it down under the LDT Rule [21]. As with all court judgements, the issue may be subject to further court proceedings. The ASCP and the College of American Pathologists (CAP) have also urged the US president to rescind the law.

## The need to advance homebrew AI systems

Given that homebrew AI systems are already permitted under existing regulatory frameworks in the EU, there is a pressing need to advance their development and integration into clinical practice. This advancement requires both utilizing existing regulatory provisions and actively promoting several key aspects of implementation. Pathologists need resources and training to develop, locally validate, calibrate, and monitor AI models within their laboratories. This effort demands collaboration between academic institutions, pathology departments, and professional organizations to share best practices and resources. Furthermore, robust and portable validation protocols and quality management systems must be established to ensure the safety and effectiveness of homebrew AI models.

The availability of open‐source AI models presents an important opportunity to accelerate this advancement. Pathology laboratories can use open‐source code and frameworks to develop customized AI models tailored to their specific needs, calibrated and validated for their local tissue preparation and staining protocols. However, integrating open‐source AI models into homebrew tests requires careful attention to legal and regulatory obligations. When using open‐source models, laboratories must meet specific requirements. The laboratory should adopt and use these models in a way that establishes them as the true ‘manufacturer’ (i.e. developer) of the model. This involves substantial adaptation through retraining, validation for local tissue preparation and imaging approaches, consideration of the local target population and indications, and ongoing monitoring to prevent model degradation due to ‘drift’. Appropriate local quality assurance processes must be maintained throughout. Additionally, laboratories must ensure complete transparency regarding the model's performance characteristics and limitations.

## Conclusion

In summary, noncommercial homebrew AI systems offer substantial potential to enhance diagnostic accuracy, efficiency, and patient outcomes in digital histopathology. Recognizing that such systems are already permitted under current EU regulatory frameworks, we advocate for their advancement and integration into pathology practice. Tighter rules that will limit the development and use of these systems in the US have been justified on the basis of promoting patient safety, but will forgo the advantages of offering pathologists medical and scientific freedom. We believe that it is better to continue to foster an environment where pathologists have the expertise to develop, validate, and supervise these models, ensuring their safe and effective use.

The time to advance homebrew AI models in pathology is now. These tools can bring cutting‐edge diagnostics to patients who would otherwise be left behind by commercial development, especially those with rare diseases. Regulatory bodies must step up to provide clear, practical guidance that enables pathologists to develop and implement these systems safely. We call on regulatory agencies, professional organizations, and pathology departments to work together with urgency. The frameworks we create today will determine whether patients worldwide can benefit from AI advances in pathology tomorrow.

## Author contributions statement

All authors conceptualized and wrote this article and agreed with its submission.

## Data Availability

Data sharing is not applicable to this article, as no datasets were generated or analysed during the current study.
